# Supplementary material for: Pyruvate Dehydrogenase Kinase 1 inhibition mediated oxidative phosphorylation enhancement in cartilage promotes osteoarthritis progression
Source: BMC Musculoskelet Disord. 2023 Jul 20;24:597. doi: 10.1186/s12891-023-06585-6 (PMC10357736; doi:10.1186/s12891-023-06585-6)
Supplement: Supplementary file 1 — Additional file 1. [file 12891_2023_6585_MOESM1_ESM.zip › BP.Gsea.1653623667859/GOBP_TRNA_PROCESSING.html]

Details for gene set GOBP\_TRNA\_PROCESSING[GSEA]

|  || Dataset | 55457\_collapsed\_to\_symbols.cls.cls #OA\_versus\_normal.cls.cls #OA\_versus\_normal\_repos |
| Phenotype | cls.cls#OA\_versus\_normal\_repos |
| Upregulated in class | OA |
| GeneSet | GOBP\_TRNA\_PROCESSING |
| Enrichment Score (ES) | 0.33400542 |
| Normalized Enrichment Score (NES) | 1.4915324 |
| Nominal p-value | 0.012658228 |
| FDR q-value | 0.39072916 |
| FWER p-Value | 1.0 |
Table: GSEA Results Summary

  

Fig 1: Enrichment plot: GOBP\_TRNA\_PROCESSING      
 Profile of the Running ES Score & Positions of GeneSet Members on the Rank Ordered List

  

| SYMBOL | TITLE | RANK IN GENE LIST | RANK METRIC SCORE | RUNNING ES | CORE ENRICHMENT || 1 | DALRD3 | DALR anticodon binding domain containing 3 [Source:HGNC Symbol;Acc:HGNC:25536] | 71 | 0.957 | 0.0289 | Yes |
| 2 | RTCB | "RNA 2',3'-cyclic phosphate and 5'-OH ligase [Source:HGNC Symbol;Acc:HGNC:26935]" | 78 | 0.947 | 0.0625 | Yes |
| 3 | ELP4 | elongator acetyltransferase complex subunit 4 [Source:HGNC Symbol;Acc:HGNC:1171] | 81 | 0.940 | 0.0962 | Yes |
| 4 | TRMT5 | tRNA methyltransferase 5 [Source:HGNC Symbol;Acc:HGNC:23141] | 125 | 0.879 | 0.1244 | Yes |
| 5 | RPP30 | ribonuclease P/MRP subunit p30 [Source:HGNC Symbol;Acc:HGNC:17688] | 265 | 0.777 | 0.1415 | Yes |
| 6 | POP5 | "POP5 homolog, ribonuclease P/MRP subunit [Source:HGNC Symbol;Acc:HGNC:17689]" | 494 | 0.662 | 0.1476 | Yes |
| 7 | SARS1 | seryl-tRNA synthetase 1 [Source:HGNC Symbol;Acc:HGNC:10537] | 516 | 0.654 | 0.1694 | Yes |
| 8 | TYW1 | tRNA-yW synthesizing protein 1 homolog [Source:HGNC Symbol;Acc:HGNC:25598] | 523 | 0.650 | 0.1924 | Yes |
| 9 | POLR3K | RNA polymerase III subunit K [Source:HGNC Symbol;Acc:HGNC:14121] | 651 | 0.605 | 0.2042 | Yes |
| 10 | CLP1 | cleavage factor polyribonucleotide kinase subunit 1 [Source:HGNC Symbol;Acc:HGNC:16999] | 748 | 0.582 | 0.2177 | Yes |
| 11 | RPP14 | ribonuclease P/MRP subunit p14 [Source:HGNC Symbol;Acc:HGNC:30327] | 835 | 0.559 | 0.2311 | Yes |
| 12 | DUS4L | dihydrouridine synthase 4 like [Source:HGNC Symbol;Acc:HGNC:21517] | 862 | 0.552 | 0.2489 | Yes |
| 13 | LCMT2 | leucine carboxyl methyltransferase 2 [Source:HGNC Symbol;Acc:HGNC:17558] | 946 | 0.529 | 0.2614 | Yes |
| 14 | PRORP | protein only RNase P catalytic subunit [Source:HGNC Symbol;Acc:HGNC:19958] | 1013 | 0.515 | 0.2748 | Yes |
| 15 | POP4 | "POP4 homolog, ribonuclease P/MRP subunit [Source:HGNC Symbol;Acc:HGNC:30081]" | 1015 | 0.514 | 0.2932 | Yes |
| 16 | POP7 | "POP7 homolog, ribonuclease P/MRP subunit [Source:HGNC Symbol;Acc:HGNC:19949]" | 1337 | 0.452 | 0.2844 | Yes |
| 17 | TRMT112 | tRNA methyltransferase activator subunit 11-2 [Source:HGNC Symbol;Acc:HGNC:26940] | 1359 | 0.449 | 0.2989 | Yes |
| 18 | WDR6 | WD repeat domain 6 [Source:HGNC Symbol;Acc:HGNC:12758] | 1373 | 0.447 | 0.3140 | Yes |
| 19 | OSGEPL1 | O-sialoglycoprotein endopeptidase like 1 [Source:HGNC Symbol;Acc:HGNC:23075] | 1593 | 0.415 | 0.3118 | Yes |
| 20 | URM1 | ubiquitin related modifier 1 [Source:HGNC Symbol;Acc:HGNC:28378] | 1605 | 0.413 | 0.3258 | Yes |
| 21 | FARS2 | "phenylalanyl-tRNA synthetase 2, mitochondrial [Source:HGNC Symbol;Acc:HGNC:21062]" | 1686 | 0.401 | 0.3340 | Yes |
| 22 | TRMT11 | tRNA methyltransferase 11 homolog [Source:HGNC Symbol;Acc:HGNC:21080] | 1885 | 0.374 | 0.3320 | No |
| 23 | MTO1 | mitochondrial tRNA translation optimization 1 [Source:HGNC Symbol;Acc:HGNC:19261] | 2181 | 0.340 | 0.3212 | No |
| 24 | FTSJ1 | FtsJ RNA 2'-O-methyltransferase 1 [Source:HGNC Symbol;Acc:HGNC:13254] | 2664 | 0.284 | 0.2938 | No |
| 25 | ELP5 | elongator acetyltransferase complex subunit 5 [Source:HGNC Symbol;Acc:HGNC:30617] | 2920 | 0.259 | 0.2832 | No |
| 26 | ELP3 | elongator acetyltransferase complex subunit 3 [Source:HGNC Symbol;Acc:HGNC:20696] | 2974 | 0.253 | 0.2882 | No |
| 27 | ELAC1 | elaC ribonuclease Z 1 [Source:HGNC Symbol;Acc:HGNC:14197] | 3108 | 0.239 | 0.2864 | No |
| 28 | DUS2 | dihydrouridine synthase 2 [Source:HGNC Symbol;Acc:HGNC:26014] | 3182 | 0.233 | 0.2891 | No |
| 29 | TRMT12 | tRNA methyltransferase 12 homolog [Source:HGNC Symbol;Acc:HGNC:26091] | 3208 | 0.230 | 0.2954 | No |
| 30 | OSGEP | O-sialoglycoprotein endopeptidase [Source:HGNC Symbol;Acc:HGNC:18028] | 3269 | 0.224 | 0.2988 | No |
| 31 | DDX1 | DEAD-box helicase 1 [Source:HGNC Symbol;Acc:HGNC:2734] | 3305 | 0.221 | 0.3040 | No |
| 32 | NSUN3 | NOP2/Sun RNA methyltransferase 3 [Source:HGNC Symbol;Acc:HGNC:26208] | 3612 | 0.192 | 0.2870 | No |
| 33 | TSEN34 | tRNA splicing endonuclease subunit 34 [Source:HGNC Symbol;Acc:HGNC:15506] | 3617 | 0.192 | 0.2936 | No |
| 34 | RTRAF | "RNA transcription, translation and transport factor [Source:HGNC Symbol;Acc:HGNC:23169]" | 3752 | 0.181 | 0.2897 | No |
| 35 | TRMT1L | tRNA methyltransferase 1 like [Source:HGNC Symbol;Acc:HGNC:16782] | 4007 | 0.155 | 0.2755 | No |
| 36 | LSM6 | "LSM6 homolog, U6 small nuclear RNA and mRNA degradation associated [Source:HGNC Symbol;Acc:HGNC:17017]" | 4019 | 0.154 | 0.2801 | No |
| 37 | HSD17B10 | hydroxysteroid 17-beta dehydrogenase 10 [Source:HGNC Symbol;Acc:HGNC:4800] | 4060 | 0.150 | 0.2824 | No |
| 38 | RPP21 | ribonuclease P/MRP subunit p21 [Source:HGNC Symbol;Acc:HGNC:21300] | 4269 | 0.131 | 0.2709 | No |
| 39 | THG1L | tRNA-histidine guanylyltransferase 1 like [Source:HGNC Symbol;Acc:HGNC:26053] | 4417 | 0.120 | 0.2637 | No |
| 40 | ELAC2 | elaC ribonuclease Z 2 [Source:HGNC Symbol;Acc:HGNC:14198] | 4532 | 0.110 | 0.2588 | No |
| 41 | GRSF1 | G-rich RNA sequence binding factor 1 [Source:HGNC Symbol;Acc:HGNC:4610] | 4660 | 0.099 | 0.2525 | No |
| 42 | ALKBH1 | "alkB homolog 1, histone H2A dioxygenase [Source:HGNC Symbol;Acc:HGNC:17911]" | 4765 | 0.090 | 0.2476 | No |
| 43 | TPRKB | TP53RK binding protein [Source:HGNC Symbol;Acc:HGNC:24259] | 5155 | 0.059 | 0.2194 | No |
| 44 | TRMT9B | tRNA methyltransferase 9B (putative) [Source:HGNC Symbol;Acc:HGNC:26725] | 5702 | 0.016 | 0.1773 | No |
| 45 | AARS1 | alanyl-tRNA synthetase 1 [Source:HGNC Symbol;Acc:HGNC:20] | 5703 | 0.015 | 0.1779 | No |
| 46 | METTL8 | "methyltransferase 8, methylcytidine [Source:HGNC Symbol;Acc:HGNC:25856]" | 5706 | 0.015 | 0.1783 | No |
| 47 | THUMPD2 | THUMP domain containing 2 [Source:HGNC Symbol;Acc:HGNC:14890] | 5913 | 0.001 | 0.1622 | No |
| 48 | LAGE3 | L antigen family member 3 [Source:HGNC Symbol;Acc:HGNC:26058] | 6180 | -0.019 | 0.1422 | No |
| 49 | TRDMT1 | tRNA aspartic acid methyltransferase 1 [Source:HGNC Symbol;Acc:HGNC:2977] | 6204 | -0.021 | 0.1411 | No |
| 50 | TRMT61B | tRNA methyltransferase 61B [Source:HGNC Symbol;Acc:HGNC:26070] | 6223 | -0.022 | 0.1405 | No |
| 51 | POP1 | "POP1 homolog, ribonuclease P/MRP subunit [Source:HGNC Symbol;Acc:HGNC:30129]" | 6261 | -0.025 | 0.1385 | No |
| 52 | TSEN2 | tRNA splicing endonuclease subunit 2 [Source:HGNC Symbol;Acc:HGNC:28422] | 6331 | -0.031 | 0.1343 | No |
| 53 | ELP1 | elongator acetyltransferase complex subunit 1 [Source:HGNC Symbol;Acc:HGNC:5959] | 6475 | -0.040 | 0.1245 | No |
| 54 | METTL2B | "methyltransferase 2B, methylcytidine [Source:HGNC Symbol;Acc:HGNC:18272]" | 6522 | -0.044 | 0.1225 | No |
| 55 | RPP38 | ribonuclease P/MRP subunit p38 [Source:HGNC Symbol;Acc:HGNC:30329] | 6814 | -0.064 | 0.1021 | No |
| 56 | KARS1 | lysyl-tRNA synthetase 1 [Source:HGNC Symbol;Acc:HGNC:6215] | 6837 | -0.066 | 0.1028 | No |
| 57 | TRMT2B | tRNA methyltransferase 2 homolog B [Source:HGNC Symbol;Acc:HGNC:25748] | 6855 | -0.068 | 0.1039 | No |
| 58 | ADAT1 | adenosine deaminase tRNA specific 1 [Source:HGNC Symbol;Acc:HGNC:228] | 7396 | -0.110 | 0.0657 | No |
| 59 | MOCS3 | molybdenum cofactor synthesis 3 [Source:HGNC Symbol;Acc:HGNC:15765] | 7752 | -0.138 | 0.0430 | No |
| 60 | CDKAL1 | CDK5 regulatory subunit associated protein 1 like 1 [Source:HGNC Symbol;Acc:HGNC:21050] | 7761 | -0.138 | 0.0473 | No |
| 61 | THADA | THADA armadillo repeat containing [Source:HGNC Symbol;Acc:HGNC:19217] | 7860 | -0.145 | 0.0449 | No |
| 62 | TARBP1 | TAR (HIV-1) RNA binding protein 1 [Source:HGNC Symbol;Acc:HGNC:11568] | 8091 | -0.163 | 0.0328 | No |
| 63 | RPP40 | ribonuclease P/MRP subunit p40 [Source:HGNC Symbol;Acc:HGNC:20992] | 8318 | -0.179 | 0.0216 | No |
| 64 | TRMO | tRNA methyltransferase O [Source:HGNC Symbol;Acc:HGNC:30967] | 8541 | -0.197 | 0.0114 | No |
| 65 | THUMPD1 | THUMP domain containing 1 [Source:HGNC Symbol;Acc:HGNC:23807] | 8561 | -0.198 | 0.0170 | No |
| 66 | QTRT1 | queuine tRNA-ribosyltransferase catalytic subunit 1 [Source:HGNC Symbol;Acc:HGNC:23797] | 8620 | -0.202 | 0.0198 | No |
| 67 | PTCD1 | pentatricopeptide repeat domain 1 [Source:HGNC Symbol;Acc:HGNC:22198] | 8674 | -0.206 | 0.0231 | No |
| 68 | TRMU | tRNA mitochondrial 2-thiouridylase [Source:HGNC Symbol;Acc:HGNC:25481] | 8916 | -0.226 | 0.0124 | No |
| 69 | WDR4 | WD repeat domain 4 [Source:HGNC Symbol;Acc:HGNC:12756] | 9631 | -0.286 | -0.0330 | No |
| 70 | METTL1 | "methyltransferase 1, tRNA methylguanosine [Source:HGNC Symbol;Acc:HGNC:7030]" | 10333 | -0.354 | -0.0750 | No |
| 71 | DTWD1 | DTW domain containing 1 [Source:HGNC Symbol;Acc:HGNC:30926] | 10549 | -0.378 | -0.0782 | No |
| 72 | TRMT61A | tRNA methyltransferase 61A [Source:HGNC Symbol;Acc:HGNC:23790] | 10603 | -0.384 | -0.0685 | No |
| 73 | RPP25 | ribonuclease P and MRP subunit p25 [Source:HGNC Symbol;Acc:HGNC:30361] | 10621 | -0.385 | -0.0560 | No |
| 74 | CDK5RAP1 | CDK5 regulatory subunit associated protein 1 [Source:HGNC Symbol;Acc:HGNC:15880] | 10870 | -0.413 | -0.0605 | No |
| 75 | NAT10 | N-acetyltransferase 10 [Source:HGNC Symbol;Acc:HGNC:29830] | 10998 | -0.429 | -0.0550 | No |
| 76 | QTRT2 | queuine tRNA-ribosyltransferase accessory subunit 2 [Source:HGNC Symbol;Acc:HGNC:25771] | 11420 | -0.489 | -0.0702 | No |
| 77 | PUS7 | pseudouridine synthase 7 [Source:HGNC Symbol;Acc:HGNC:26033] | 11421 | -0.490 | -0.0526 | No |
| 78 | PUS3 | pseudouridine synthase 3 [Source:HGNC Symbol;Acc:HGNC:25461] | 11503 | -0.501 | -0.0409 | No |
| 79 | TRIT1 | tRNA isopentenyltransferase 1 [Source:HGNC Symbol;Acc:HGNC:20286] | 11658 | -0.526 | -0.0340 | No |
| 80 | TRMT1 | tRNA methyltransferase 1 [Source:HGNC Symbol;Acc:HGNC:25980] | 11775 | -0.545 | -0.0234 | No |
| 81 | TRMT13 | tRNA methyltransferase 13 homolog [Source:HGNC Symbol;Acc:HGNC:25502] | 11792 | -0.548 | -0.0050 | No |
| 82 | SSB | small RNA binding exonuclease protection factor La [Source:HGNC Symbol;Acc:HGNC:11316] | 11955 | -0.584 | 0.0034 | No |
| 83 | GTPBP3 | "GTP binding protein 3, mitochondrial [Source:HGNC Symbol;Acc:HGNC:14880]" | 11993 | -0.592 | 0.0218 | No |
| 84 | PUS1 | pseudouridine synthase 1 [Source:HGNC Symbol;Acc:HGNC:15508] | 12260 | -0.664 | 0.0249 | No |
| 85 | DUS1L | dihydrouridine synthase 1 like [Source:HGNC Symbol;Acc:HGNC:30086] | 12355 | -0.693 | 0.0425 | No |
Table: GSEA details [plain text format]

  

Fig 2: GOBP\_TRNA\_PROCESSING      
 Blue-Pink O' Gram in the Space of the Analyzed GeneSet

  

Fig 3: GOBP\_TRNA\_PROCESSING: Random ES distribution      
 Gene set null distribution of ES for **GOBP\_TRNA\_PROCESSING**
